# Characterization of bioactive compounds in fenugreek genotypes in varying environments: diosgenin, trigonelline, and 4-hydroxyisoleucine

**DOI:** 10.3389/fpls.2025.1562931

**Published:** 2025-03-24

**Authors:** Furkan Coban, Hakan Ozer, Bilal Yilmaz, Yuzhou Lan

**Affiliations:** ^1^ Department of Plant Breeding, The Swedish University of Agricultural Sciences, Lomma, Sweden; ^2^ Department of Field Crops, Faculty of Agriculture, Ataturk University, Erzurum, Türkiye; ^3^ Department of Analytical Chemistry, Faculty of Pharmacy, Ataturk University, Erzurum, Türkiye

**Keywords:** diosgenin, trigonelline, 4-hydroxyisoleucine, fenugreek, genetic background

## Abstract

This study investigates the effects of irrigated and non-irrigated conditions on the bioactive compound content in fenugreek (*Trigonella foenum-graecum*) across 31 diverse genotypes from various geographical regions. The study was conducted at Atatürk University Research and Extension Center, Türkiye (N 39°55’59.9”, E 41°14’10.6”, altitude 1789 m) during the 2021 and 2022 growing seasons. The levels of diosgenin, trigonelline, and 4-hydroxyisoleucine analyzed under irrigated and non-irrigated conditions were found to be significantly influenced by genotype, environment, and their interaction (Genotype × Environment), with a highly significant effect observed at the p < 0.001 level. The compounds analyzed included diosgenin (0.50-0.93%), trigonelline (5.22-13.65 mg g^-^¹), and 4-hydroxyisoleucine (0.41-1.90%). Notably, genotypes such as Sivas/TR, Amasya/TR, Konya/TR and Samsun/TR exhibited higher diosgenin content across all conditions, while Spain, Malaysia, France, and India showed higher trigonelline content under irrigation. Variability in 4-hydroxyisoleucine content was observed, with some genotypes showing stability across different environmental conditions. A negative correlation between diosgenin and trigonelline was observed in fenugreek. Furthermore, Principal Component Analysis (PCA) and cluster analysis were found to be effective in revealing genetic diversity, morphological differences, and genotype adaptability. The findings highlight the potential for selecting superior genotypes for breeding programs focused on enhancing bioactive compound yields, especially under varying irrigation and non-irrigated conditions. This research emphasizes the critical role of environmental and genetic factors in optimizing the production of health-benefiting compounds in fenugreek.

## 1 Introduction

**Figure 1:**
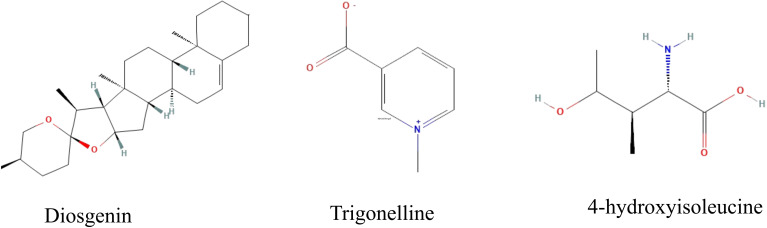
Chemical structures of diosgenin, trigonelline, and 4-hydroxyisoleucine.

Fenugreek (*Trigonella foenum-graecum*), widely recognized for its culinary and medicinal applications, is an annual herb belonging to the family Leguminosae (Zandi et al., 2015; Maloo et al., 2023). Fenugreek is a rich source of bioactive compounds beyond its traditional uses, particularly diosgenin, trigonelline, and 4-hydroxyisoleucine, which have demonstrated significant health benefits (**Figure 1**). Diosgenin has been recognized for its positive effects on glucose metabolism and diabetes management (Narender et al., 2006). Trigonelline plays a crucial role in reducing oxidative stress, which is a key factor in the development of cardiovascular diseases and neurodegenerative disorders (Mohammad-Sadeghipour et al., 2021). Additionally, 4-hydroxyisoleucine has been identified as a key amino acid that enhances insulin secretion, making it a promising candidate for type 2 diabetes treatment (Faisal et al., 2024). Given these bioactive compounds, fenugreek has been increasingly studied for its potential role in managing chronic diseases such as diabetes, cardiovascular diseases, and metabolic syndrome (Khorshidian et al., 2016; Nguyen et al., 2024; Tak et al., 2024).

Bioactive compounds in plants are significantly influenced by various environmental factors, including temperature, light, soil nutrients, water availability, and interactions with other organisms (Qaderi et al., 2023; Tsipinana et al., 2023). These environmental conditions lead to noticeable differences in the production of secondary metabolites (Khare et al., 2020; Fouad et al., 2023). Irrigation plays a critical role in promoting optimal plant growth, biomass production, and overall yield. Adequate water supply ensures the proper maintenance of plant physiological processes and photosynthesis, resulting in significant growth improvements (Prinsloo and Nogemane, 2018). Under sufficient irrigation (defined as 100% of the crop water requirement, ETc), plants effectively synthesize primary metabolites and, under favorable conditions, produce secondary metabolites at notable levels. In contrast, non-irrigated conditions rely solely on natural precipitation, which may not fully meet the water demands of plants, potentially limiting growth and metabolism (Isah, 2019). However, secondary metabolite levels in plants may be lower under drought stress due to the adequate water supply reducing environmental stress and suppressing the metabolic signals that trigger the production of defense-related secondary metabolites (Zarinkamar et al., 2022; Camlica and Yaldiz, 2024). On the other hand, the increase in secondary metabolites in plants irrigated before drought reaches a critical level suggests that the production of these compounds is not solely a response to stress but is also linked to the plant’s optimal metabolic functioning (Erb and Kliebenstein, 2020; Afrouz et al., 2023). Additionally, irrigation enhances primary metabolite production, thereby supporting the formation of precursor molecules required for the synthesis of secondary metabolites (Al Kashgry et al., 2024). Agricultural management practices must integrate strategies that balance irrigation and environmental stress conditions, such as drought, heat stress, and salinity, to achieve both resilience and high productivity. Climate change, characterized by rising temperatures, irregular rainfall, and prolonged droughts, directly impacts plant metabolism, emphasizing the need for robust stress tolerance mechanisms. Managing genetic and environmental factors harmoniously is crucial, especially for regulating secondary metabolite production under varying environmental conditions (Gadanakis et al., 2015; Yoon et al., 2020).

Diosgenin is a bioactive compound found in fenugreek seeds, possessing significant potential for diabetes treatment. Diosgenin mitigates the harmful effects of diabetes in rodents by reducing insulin resistance, lowering plasma glucose levels, and promoting pancreatic beta cell regeneration (Tharaheswari et al., 2014). Its antioxidant properties help reduce oxidative stress and provide cellular protection. Studies have demonstrated that diosgenin significantly decreases plasma glucose levels, increases insulin levels in diabetic rats, and inhibits cancer cell growth while inducing apoptosis (Pari et al., 2012; Hao et al., 2015; Tak et al., 2024). This compound stands out as a promising bioactive ingredient in the treatment of chronic diseases like diabetes and cancer.

Another remarkable compound, trigonelline (TRG) is a naturally occurring alkaloid with promising therapeutic potential due to its multifaceted pharmacological properties. TRG has been recognized for its potential in managing metabolic, inflammatory, and oxidative stress-related conditions; (Nguyen et al., 2024). TRG can modulate glucose and lipid metabolism, making it beneficial for patients with diabetes and obesity (Hamden et al., 2013; Yoshinari et al., 2013; Ilavenil et al., 2015). Moreover, it exhibits anti-inflammatory and antioxidant properties, which are crucial in mitigating chronic inflammatory diseases and oxidative stress (Hamadi, 2012; Zhou et al., 2013; Li et al., 2019). TRG also shows neuroprotective effects, offering potential benefits in treating neurodegenerative disorders like Alzheimer’s and Parkinson’s diseases, as well as cognitive impairments and diabetic neuropathy (Makowska et al., 2014; Fahanik-Babaei et al., 2019; Farid et al., 2020; Liang et al., 2023);. Additionally, TRG has been observed to protect against liver and kidney injuries, cardiovascular diseases, and certain cancers by inhibiting tumor cell proliferation and inducing apoptosis (Afifi et al., 2017; Peerapen et al., 2023)e;. These broad-spectrum therapeutic effects underline TRG’s potential as a valuable natural compound in developing treatments for various pathological conditions (Yoshinari and Igarashi, 2010; Costa et al., 2020).

Also, another compound found in the fenugreek seed is 4-Hydroxyisoleucine (4-HIL). This amino acid exhibits notable antihyperglycemic and antihyperlipidemic properties (Narender et al., 2006). It enhances insulin secretion, thereby lowering blood glucose levels, and reduces plasma triglycerides, total cholesterol, and free fatty acids while increasing the HDL-C/TC ratio. Studies have shown that 4-HIL regulates key genes involved in lipid metabolism, in human colorectal cancer cells, indicating its potential therapeutic role in managing metabolic disorders like diabetes and dyslipidemia (Mohammad-Sadeghipour et al., 2021).

In recent years, research on the adaptation of fenugreek genotypes to environmental conditions has increased both in Türkiye and globally (Beyzi et al., 2021; Maloo et al., 2023; Camlica and Yaldiz, 2024; Camlica et al., 2024; Coban et al., 2024; Ghosaliya et al., 2024; Haliloğlu et al., 2024). Changing climate conditions, particularly drought and irregular rainfall patterns, are significant stress factors that negatively impact the growth and yield potential of fenugreek (Abd-El-Wahab et al., 2023; Dobeie et al., 2024). However, fenugreek stands out not only for its agricultural use but also for its bioactive compounds, which have important applications in the health sector. Additionally, due to its suitability for mechanization, short vegetation period, and low production cost, fenugreek is considered a more suitable alternative to plants from the Dioscorea family, traditionally used for diosgenin production (Coban, 2021). These characteristics make fenugreek a valuable resource for both sustainable agriculture and the pharmaceutical and food industries.

This study aims to provide a novel perspective on the production of significant bioactive compounds in fenugreek by investigating the impact of irrigated and non-irrigated conditions on 31 diverse genotypes collected from different regions worldwide. By combining multivariate statistical approaches, this research uniquely elucidates the complex genotype-environment interactions that influence the production of diosgenin, trigonelline, and 4-hydroxyisoleucine. Furthermore, it identifies the regions and genotypes with the highest bioactive compound content, offering valuable insights for the selection of superior genotypes in future breeding programs. This comprehensive evaluation not only highlights the genetic and environmental factors driving metabolite variability but also addresses the increasing demand for bioactive compound-rich crops in the context of climate change and sustainable agriculture.

## 2 Materials and methods

### 2.1 Plant materials

In this research, 31 distinct fenugreek seed genotypes were obtained from diverse locations, including Iran (IR), Türkiye (TR), Egypt, Germany, South Sudan, France, Australia, Spain, Morocco, Ukraine, China, Serbia, Israel, Malaysia, Pakistan, and India (**Table 1**). Berkem, Çiftçi, and Güraslan are genotypes that were previously registered by the Seed Registration and Certification Center Directorate of Türkiye. The seeds were obtained from research centers, international seed banks, field projects, local vendors, and local producers. The classification of fenugreek seed genotypes into Group A and Group B was primarily based on observable phenotypic and genotypic differences. These differences were particularly prominent in genotypes originating from Iran (IR) and Türkiye (TR). During preliminary analyses, these genotypes exhibited significantly distinct results in several key traits, such as germination rates and environmental adaptability. These remarkable regional variations led to the inclusion of genotypes from Iran and Türkiye in Group B to facilitate a more detailed analysis of their unique characteristics. In contrast, genotypes obtained from other regions (Group A) displayed more homogeneous traits, aligning with broader global patterns. In this context, the classification of genotypes in this manner aimed not only to highlight the distinctiveness of Iranian and Turkish genotypes but also to provide a clearer framework for comparing regional and global trends observed in the study. Accession numbers are also listed in **Table 1**.

**Table 1:** Origin, accession numbers and group classification of fenugreek genotypes.

Origin	Accessions	Group	Accession Type
Urmia/IR	ZFTB0001	B	Population
Yozgat/TR	ZFTB0002	B	Population
Samsun/TR	ZFTB0003	B	Population
Sanliurfa/TR	ZFTB0004	B	Population
Sivas/TR	ZFTB0005	B	Population
Corum/TR	ZFTB0006	B	Population
Egypt	ZFTB0007	A	Population
Germany	ZFTB0009	A	Population
Kermanshah/IR	ZFTB0010	B	Population
Berkem/TR	ZFTB0011	B	Cultivar
South Sudan	ZFTB0012	A	Population
Güraslan/TR	ZFTB0013	B	Cultivar
France	ZFTB0014	A	Population
Australia	ZFTB0015	A	Population
Spain	ZFTB0016	A	Population
Morocco	ZFTB0017	A	Population
Ukraine	ZFTB0018	A	Population
China	ZFTB0019	A	Population
Salmas/IR	ZFTB0020	B	Population
Kayseri/TR	ZFTB0021	B	Population
Serbia	ZFTB0022	A	Population
Israel	ZFTB0023	A	Population
Tokat/TR	ZFTB0024	B	Population
Malaysia	ZFTB0026	A	Population
Konya/TR	ZFTB0027	B	Population
Karaman/TR	ZFTB0028	B	Population
Ahvaz/IR	ZFTB0029	B	Population
Amasya/TR	ZFTB0030	B	Population
Pakistan	ZFTB0031	A	Population
India	ZFTB0034	A	Population
Çiftçi/TR	ZFTB0035	B	Cultivar

“TR” represents Türkiye, and “IR” represents Iran.

### 2.2 Site and experiment description

The field experiment was conducted at Ataturk University Research and Extension Center (39°55’59.9”N, 41°14’10.6”E) in Türkiye, located at an altitude of 1789 m. The study was laid out in a Randomized Complete Block Design (RCBD) with three replications. The experimental plots, measuring 5.0 m x 1.2 m, were sown with spring wheat as the previous crop in both years. Fenugreek seeds were sown at a rate of 40 kg ha^-1^ with a row spacing of 30 cm. Fenugreek seeds were sown at a rate of 40 kgha-1 with a row spacing of 30 cm (**Supplementary Video 1**). Irrigation was performed during the flowering and seed formation stages, when the water demand of fenugreek plants increases, using the furrow irrigation method. Groundwater was used for irrigation, supplied from an irrigation pond located near the experimental area. The irrigation water was classified as high-quality water with no salinity or sodium-related issues. A total of five irrigation applications were carried out in 2021, whereas four irrigation applications were performed in 2022 to fulfill the water requirements of fenugreek. After harvesting, the seeds were stored under appropriate conditions to maintain their quality. To prevent the deterioration of genetic material, the seeds were preserved at 4°C with a moisture content of 8-10%. These conditions are considered ideal for ensuring the homogeneity of the genotypes and the accuracy of subsequent analyses. **Table 2** presents detailed information about the research. Field trials were established as separate experiments under irrigated and non-irrigated conditions.

**Table 2:** Site and experimental design details.

Site Coordinates	N 39°55’59.9” E 41°14’10.6”	
*Location*	Ataturk University Research and Extension Center/Türkiye	
*Altitude (m)*	1789	
*Study design*	Randomized Complete Blok Design (RCBD-three replications)	
*Previous crop*	Spring wheat (both year)	
*Plot size*	5.0 m x 1.2 m	
*Rows* sp*ace*	30 cm	
*Sowing rate*	40 kg ha^-1^	
*Sowing method*	Plot drill (Pocta, Model CP-1 SR-1)	
*Nitrogen fertilezer, IR*	40 kg N ha^-1^ (AS^*^)	
*Phosphor fertilezer, IR*	60 kg N ha^-1^ (TSP^**^)	
*Nitrogen fertilezer, Non-IR*	20 kg N ha^-1^ (AS)	
*Phosphor fertilezer, Non-IR*	30 kg N ha^-1^ (TSP)	
Soil texture^***^	Clay-loam	
*Soil pH (0-30 cm depth)*	7.45	
*CaCO_3_ (%)*	0.795	
*Organic matter (%)*	1.29	
*P_2_O_5_ (kg/ha)*	77.3	
*K_2_O (kg/ha)*	1633.3	
	2021	2022
*Irrigation Management* ^&^	5x	4x
*Sowing Date*	6 May	6 May
*Vegetation Day*	108-132	101-138

^*^AS, ammonium sulfate; ^**^TSP, triple superphosphate; ^***^The soil properties are provided according to the average of the years. ^&^Specified for irrigated conditions.

It was observed that the total rainfall in the first year was lower than the average of the second year and long-term average. In 2021, the lowest rainfall was recorded in May and June (3.8 mm and 2.4 mm, respectively), which are critical months for plant emergence and growth, while rainfall increased in July and August. In contrast, in 2022, rainfall in May and June (41.0 mm and 65.2 mm, respectively) was significantly higher, but it dropped to very low levels in July and August. The average temperatures in 2021 and 2022 were similar. Particularly in 2021, the combination of low rainfall during critical growth periods and high temperatures highlighted an increased risk of drought (**Table 3**). Indeed, 2021 was recorded as the driest year in Türkiye in the last 20 years (Soylu Pekpostalci et al., 2023). Due to germination and emergence problems in trials conducted under non-irrigated conditions, data could not be collected homogeneously, and reliable results could not be obtained. Therefore, the dry conditions of 2021 were excluded from the study. Based on this, the research continued under the following conditions: 2021-irrigated, 2022-non-irrigated, and 2022-irrigated. It has been determined that the soils have a slightly alkaline character, with low levels of total nitrogen, available phosphorus, and lime, very low levels of organic matter, and, on the other hand, are rich in plant-available potassium.

**Table 3:** Climate data (temperature, precipitation, and relative humidity) for the vegetation period in the research area, encompassing long-term averages (1990–2020) along with sowing and harvest times for the years 2021 and 2022.

Years	Months					Annual precipitation (mm)/ Average temperature (°C) and humidity (%)
	May	June	July	August	September	
Precipitation (mm)						
*1990–2020*	52,3	41,0	25,2	16,7	4,32	139,5
*2021*	3,8	7,4	30,3	38,6	5,85	86,0
*2022*	41,0	65,2	3,10	8,10	0,30	117,7
Temperature (°C)						
*1990-2020*	10,5	14,9	19,2	19,4	14,1	15,6
*2021*	18,0	17,6	20,7	19,9	13,2	17,9
*2022*	10,9	16,7	20,5	23,3	21,6	18,6
Humidity (%)						
*1990-2020*	64,1	58,9	53,3	49,8	52,6	55,7
*2021*	36,3	43,3	47,7	48,6	45,6	44,3
*2022*	60,4	60,8	48,6	39,8	38,7	49,6

The data covers the months of May (May 6-31), June, July, August, and September (September 1-10).

### 2.3 Determination of bioactive compounds

#### 2.3.1 Determination of diosgenin (%)

Diosgenin content was quantified using a modified version of the method reported by Taylor et al. (2000). In this process, 10 g of dried and powdered fenugreek seeds were first defatted using petroleum ether (Merck, Darmstadt, Germany) in a Soxhlet apparatus (Isolab, Wertheim, Germany) for 6 hours. After filtering and drying the material, an extract was obtained with 300 mL of 80% ethanol (Sigma-Aldrich) using a rotary evaporator (Heidolph Instruments GmbH and Co.KG, Schwabach, Germany) at 80°C. After evaporating the solvent, the remaining extract was hydrolyzed with 120 mL of 70% isopropanol (Thermo Fisher Scientific, Bridgewater, NJ, USA) containing 1M H_2_SO_4_ (Merck) at 100°C for 2 hours. The solvent was evaporated again, and 90 mL of hexane (Sigma-Aldrich) was added to the remaining extract. The resulting extract was washed 3 times with 30 mL of 4N NaOH (Merck) and then 3 times with 30 mL of distilled water, after which the hexane was evaporated. The extracts were dissolved in 1 mL of methanol (Merck) and injected into a GC-MS system. For GC-MS analysis, an Agilent 6890N Gas Chromatography system (Agilent Technologies, Palo Alto, CA, USA) equipped with an Agilent 5973N CI/EI MS Detector and an Agilent 7673 Autosampler was used. Quantitative analysis of the prepared extracts was performed using standard diosgenin (D1634 Sigma-Aldrich Diosgenin ≥93%) to construct a calibration curve to determine the diosgenin content in the extracts. A HP-5 MS column (Agilent Technologies, dimensions: 30 m x 0.250 mm ID and 0.25 μm film thickness) was employed for separation. The splitless injection sample technique (1 μL) and a helium carrier gas at a flow rate of 1 mL/min were used. The GC oven temperature program was set to start at 200°C, held for 1.0 minutes, then increased at a rate of 10°C per minute to 290°C, and held for 1.0 minute.

In the National Institute of Standards and Technology Library Version (2005), Software, Turbomass 5.2, the range of the obscure segment was compared with the range of the part stored identification of diosgenin. By comparing direct kovats maintenance list and mass spectra with those obtained from the MS library, the pieces could be separated. Every component relative rate measure was calculated by comparing its typical pinnacle region to the total areas. The test materials component names, atomic weights, and structures were uncovered. The purity and specificity of the method was investigated by observing interferences between diosgenin and the excipients. For GC-MS, electron impact mode with selected ion monitoring (SIM) was used for quantitative analysis (m/z 139 for diosgenin).

#### 2.3.2 Determination of trigonelline (mg g^-1^)

To determine the TRG content in fenugreek seeds, we followed the method described by Shailajan et al. (2011) with slight modifications. Specifically, 10g of ground dry seed samples were diluted with 200 mL ethanol (Sigma-Aldrich) at a 1:10 ratio, mixed for 60 seconds on a vortex mixer (IKA, Staufen, Germany), and then placed on an orbital shaker (Heidolph Instruments GmbH and Co.KG, Schwabach, Germany) at 150 rpm for 12 hours. The mixture was filtered through Whatman No. 1 filter paper and stored at +4°C prior to analysis. For the standard solution (CAS Number: 6138-41-6; EC Number: 228-119-5; Sigma-Aldrich, USA), TRG (10 mg) was dissolved in 10 mL of methanol (Merck) to prepare a 1000 μg/mL stock solution. The high-performance liquid chromatography (HPLC) system (FRC-10A, Shimadzu Scientific Instruments, Japan) consisted of a diode-array detector (DAD, SPD-M20A), system control unit (LC-20ADXR), degassing unit (DGU-20ASR), pump (LC-20ADXR), column oven (CTO-10ASVP), and autosampler (SIL-20AXR). The TRG content in fenugreek seeds was quantified using a reverse-phase Inertsil ODS-3 column (5 μm, 250×4.6 mm, GL Sciences, Japan). The mobile phase consisted of methanol and ultrapure water (Milli-Q system, Millipore, Burlington, USA) at a ratio of 95:5 (v/v), adjusted to a pH of 3.5 with hydrochloric acid (HCl, Sigma-Aldrich). The analysis was carried out at 267 nm with a flow rate of 1.0 mL/min at room temperature (27 ± 1°C), and an injection volume of 10 μL was used.

#### 2.3.3 Determination of 4-hydroxyisoleucine (%)

The 4-HIL content was determined by the method used by Abd-El Mawla and Osman (2011) with slight modifications. 10g of fenugreek seeds were placed in a centrifuge tube, and 5 mL of 1.0 M sulfuric acid was added. The mixture was homogenized and incubated in a water bath at 30°C for 2 hours. Then, 5 mL of ethyl acetate was added, mixed for 10 seconds using a vortex mixer, and centrifuged (5000 rpm, 5 minutes). The organic phase was collected and evaporated under a nitrogen gas stream. Finally, the residue was dissolved in 1.0 mL methanol and 20 μL was injected into the HPLC system. The 4-HIL content was expressed as a percentage of dry matter. The analysis was performed using an HPLC system with a DAD detector. A C18 reverse-phase column (5 μm, 250×4.6 mm i.d.) was used. The mobile phase consisted of 0.1 M ammonium hydroxide (A) and acetonitrile (B), and the analysis was conducted using a gradient elution technique with a gradient of 10-30% (B) over 30 minutes. The column flow rate was set at 1.0 mL/min, and detection was performed at a wavelength of 265 nm. The injection volume was 30 μL.

### 2.4 Data analysis

Statistical analyses were conducted in Microsoft Excel (2016) and RStudio (Team, 2015). To evaluate the interaction effect of genotype and environment, a two-way analysis of variance (ANOVA) was performed., Due to the lack of normal distribution of Diosgenin and 4-Hydroxyisoleucine content among genotypes, a permutation-based approach with a permutation number of 5000 was performed to assess the statistical significance of ANOVA outcome using the R package ‘RVAideMemoire’. A pair-wise mean comparison was conducted between different environments using the posthoc LSD test with the R package ‘agricolae’. A hierarchical clustering was conducted and visualized in the form of a heatmap using the R package ‘pheatmap’. The relationship between diosgenin, TRG and 4-HIL was examined by linear regression. Principal component analysis (PCA) was conducted separately in each environment using the R package ‘ggfortify’. To assess the stability of each genotype across three environments (2021irrigated, 2022-irrigated, 2022 non-irrigated), the additive main effects and multiplicative interaction (AMMI) were computed using the R package ‘metan’.

## 3 Results

### 3.1 Genotypic variations in bioactive compounds in relation to environments

**Figure 2:**
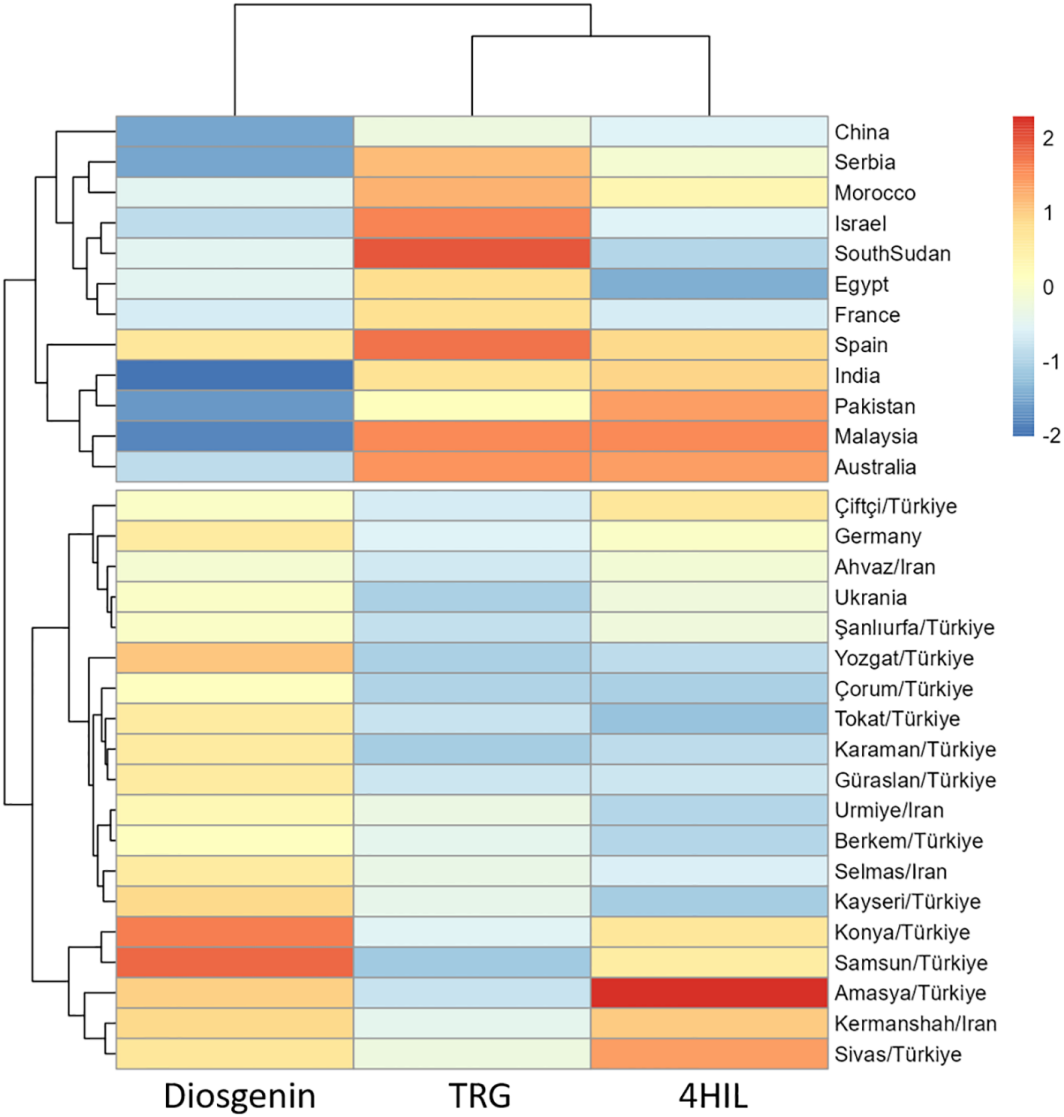
Cluster analysis of fenugreek genotypes based on diosgenin, TRG, 4-HIL.

Basically, all three bioactive compounds i.e. Diosgenin, TRG and 4-HIL varied significantly among genotypes (**Table 4**), indicating promising genetic diversity of the genotypes used in this study. The hierarchical clustering based on three bioactive compounds divided 31 genotypes into two clusters, which matched our regional grouping method (group A and B) well with only two genotypes i.e. Germany and Ukraine allocated to the cluster dominated by Iran and Türkiye genotypes (**Figure 2**). Further, the changing environments, interacting with genotypic variations showed significant impacts (p < 0.001) on these bioactive compounds (**Table 4**).

**Table 4:** ANOVA table in the form of mean square values for Diosgenin, TRG and 4-HIL investigated in different environments (***sig. < 0.001).

	Genotype	Environment	Genotype * Environment
Degree of freedom	30	2	60
Diosgenin	0.085***	0.54***	0.0033***
TRG	6.29***	64.05***	8.23***
4-HIL	0.95***	2.66***	0.16***

**Figure 3:**
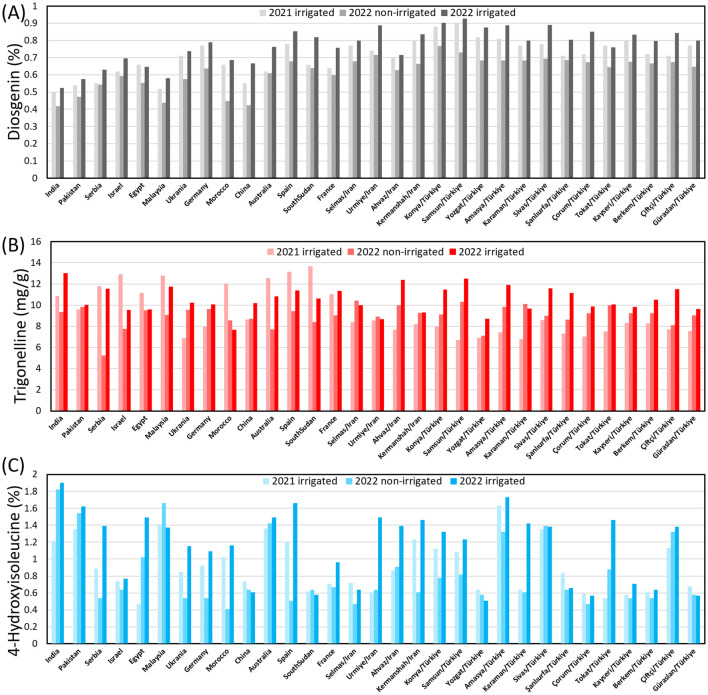
Genotypic variation in the content of **(A)** diosgenin, **(B)** TRG and **(C)** 4-HIL under three environments.

Across the three environments, the content of diosgenin, TRG and 4-HIL ranged between 0.42 - 0.93%, 5.22 - 13.65 mg g^-1^ and 0.41 - 1.90%, respectively (**Figure 3**). Under 2021-irrigated conditions, the Samsun genotype (0.90%) exhibited the highest diosgenin content, while India (0.50%) had the lowest. In 2022-non-irrigated conditions, the Konya genotype (0.77%) showed the highest diosgenin content, followed by Samsun/TR (0.73%), Urmia/IR (0.72%), and Sivas/TR (0.69%), with India again having the lowest (0.42%). Under 2022-irrigated conditions, Samsun/TR (0.93%) consistently maintained the highest diosgenin content. For 4-hydroxyisoleucine (4-HIL), under 2021-irrigated conditions, the Amasya/TR genotype (1.63%) had the highest level, while under 2022-irrigated conditions, India (1.90%) and Malaysia (1.66%) genotypes led. Trigonelline content under 2021-irrigated conditions, with South Sudan (13.65 mg g^-1^) showing the highest, while under 2022 -irrigated conditions, India recorded the highest (13.02 mg g^-1^). Across both years, genotypes grown under irrigated conditions generally exhibited higher diosgenin, 4-HIL, and trigonelline contents, highlighting the significant influence of environmental conditions on bioactive compound production. Clearly, all the Group-B genotypes exhibited close values in diosgenin and TRG while the other Group-A genotypes varied more drastically. The 4-HIL showed a larger variation among both Group-B and Group-A genotypes, indicating a strong genetic diversity in this compound (**Supplementary Table 1**).

Therefore, a mean comparison was performed between Group-A and Group-B genotypes. Interestingly, the Group-B genotypes showed significantly higher content of diosgenin than the Group-A genotypes across three environments while Group-A genotypes were found with higher content of TRG than Group-B genotypes in 2021-irrigated environment. These trends implied a clear regional pattern of the diosgenin and TRG contained in fenugreek seeds. No difference was found in average 4-HIL content between Group A and Group B genotypes.

**Figure 4:**
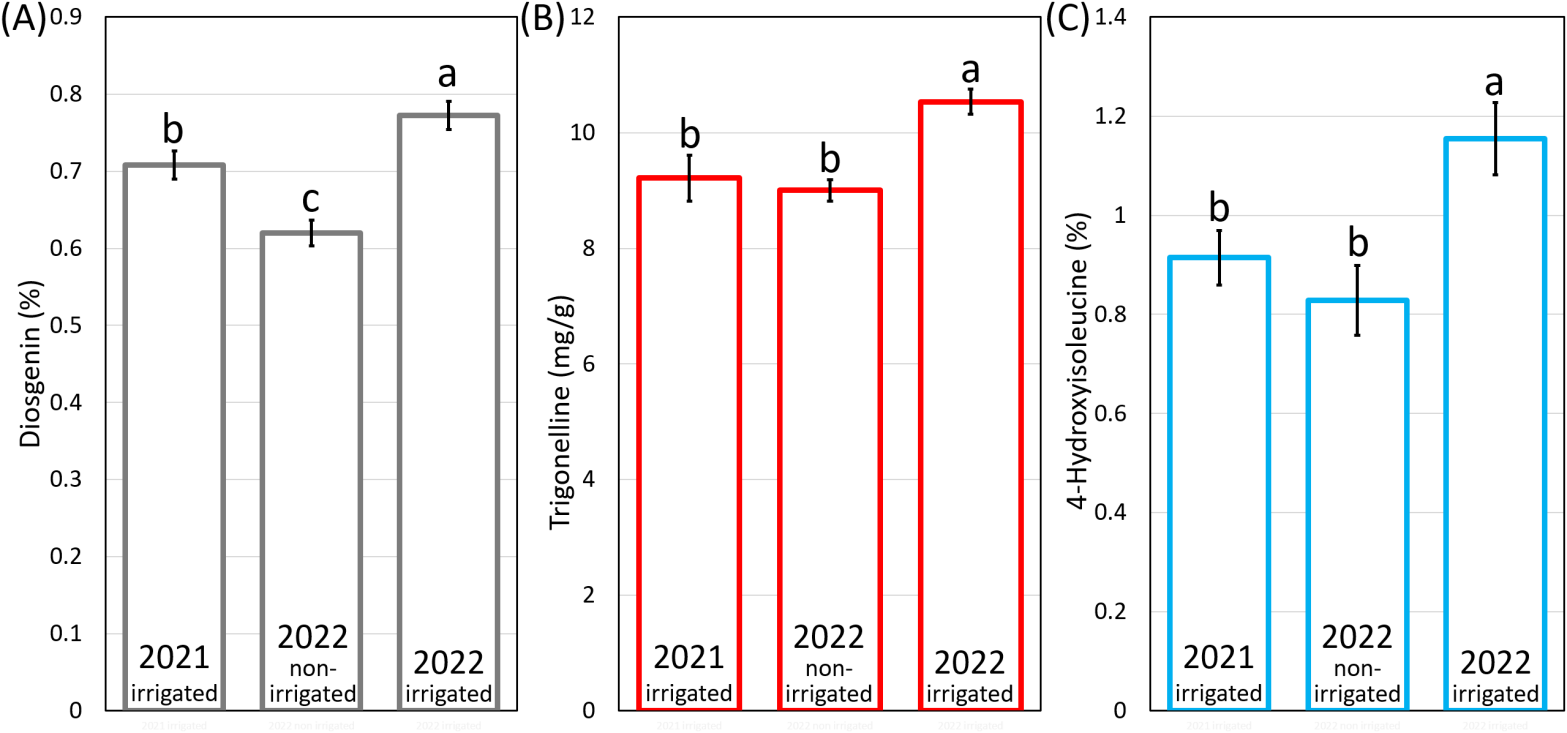
The average content of **(A)** diosgenin, **(B)** TRG and **(C)** 4-HIL under three environments. Means marked by the same letter do not differ significantly (LSD *post hoc* test at p < 0.05).

Genotypes grown in 2022-irrigated condition exhibited the significantly highest average content of diosgenin, TRG and 4-HIL across three environments (**Figure 4**). The 2022-non-irrigated environment resulted in the lowest average diosgenin content (**Figure 4A**) while no difference was found between 2022-non-irrigated and 2021-irrigated genotypes in TRG and 4-HIL content (**Figures 4B, C**).

### 3.2 Relationships between genotype and bioactive compounds in each environment

The Group-A genotypes, except for Germany and Ukraine, were all located with negative values on PC1, which primarily reflects the distribution of TRG content among genotypes with eigenvectors of -0.69 and -0.14 on PC1 and PC2 respectively. This negative assignment arises because TRG content was strongly correlated with the negative direction of PC1, as determined by the biplot analysis. PCA separates groups by maximizing variance along the first principal component (PC1), which explained 55.4% of the total variation in this analysis. In this case, the high TRG content of Group-A genotypes contributed significantly to the variation captured in the negative region of the PC1 axis. Thus, the positioning of Group-A genotypes along the negative PC1 axis clearly differentiates them from Group-B genotypes, which exhibited higher diosgenin content associated with positive PC1 values. This contrast highlights the primary traits defining each genotype group under the 2021-irrigated environment.

**Figure 5:**
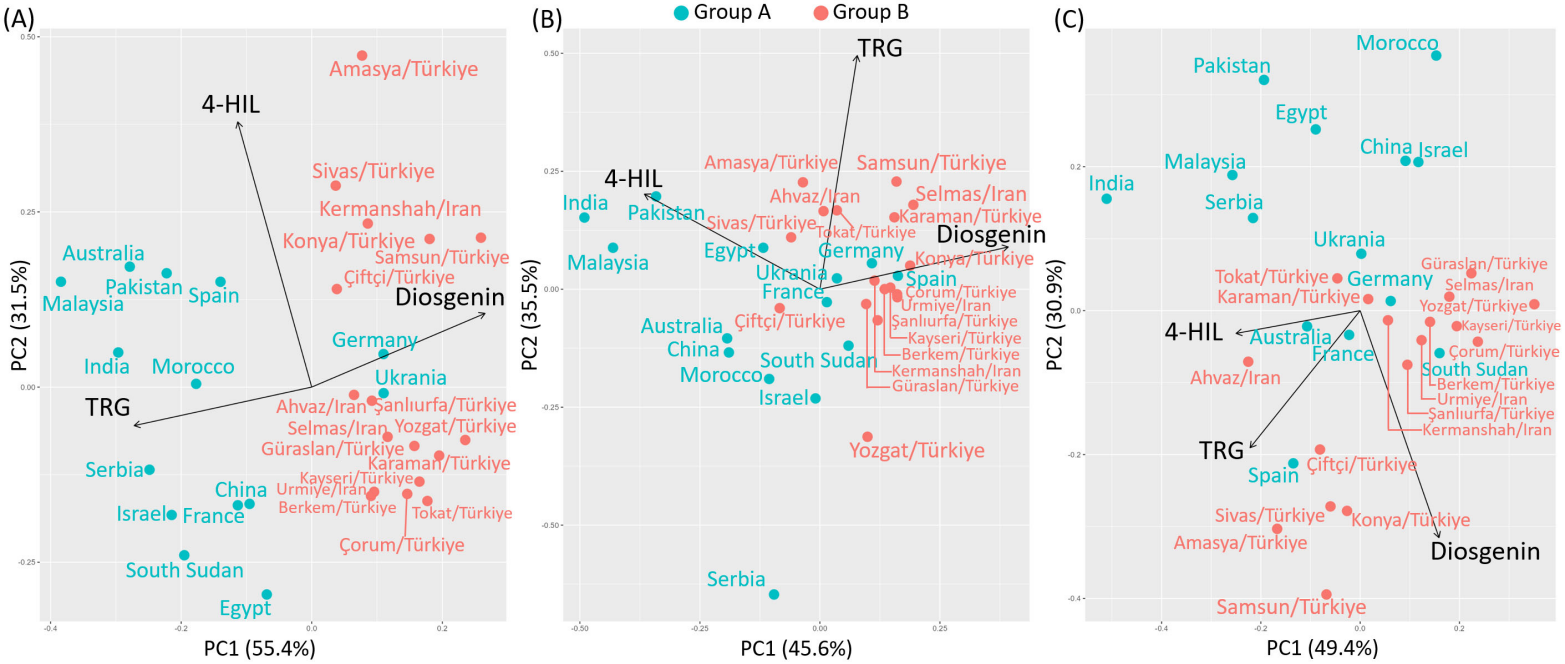
Principal component analysis (PCA) for the content of diosgenin, TRG and 4-HIL in Group A and Group B genotypes under **(A)** 2021-irrigated, **(B)** 2022-non-irrigated and **(C)** 2022-irrigated environments.

**Figure 6:**
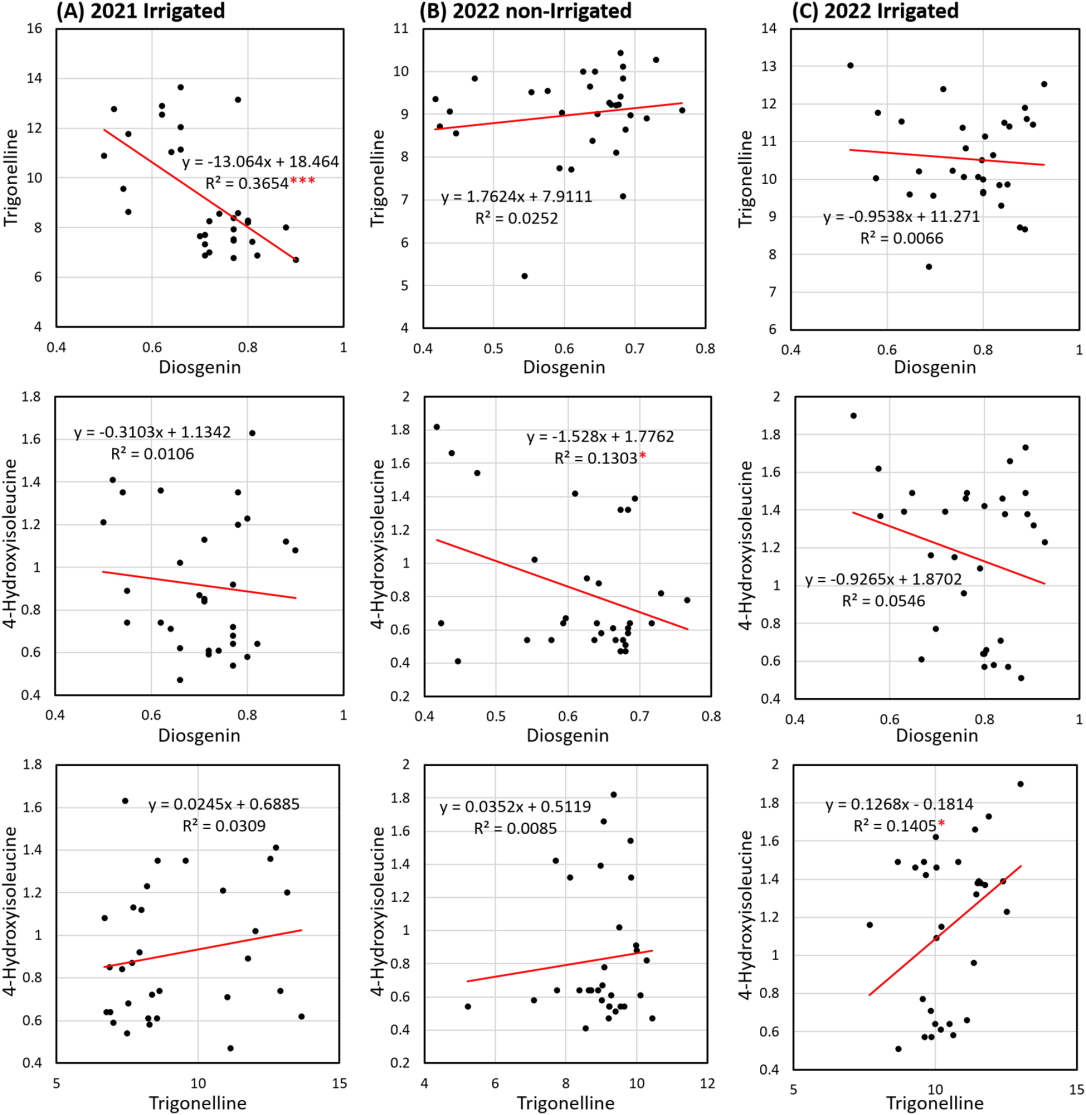
Linear regression (R2 = the coefficient of determination) of diosgenin versus TRG, diosgenin versus 4-HIL and TRG versus 4-HIL in 31 genotypes under three environments including **(A)** 2021-irrigated, **(B)** 2022-non-irrigated and **(C)** 2022-irrigated (*sig. < 0.05; ***sig. < 0.001).

The second principal component (PC2) accounting for 31.5% of the total variation mainly differentiated the variation of 4-HIL, with eigenvectors of -0.29 and 0.95 on PC1 and PC2 respectively (**Figure 5A**). Under 2022-non-irrigated environment, the Group-B genotypes maintained a relatively high diosgenin content while several Group-A genotypes e.g. India, Pakistan and Malaysia exhibited a high 4-HIL (eigenvectors on PC1 and PC2: -0.67 and 0.37) (**Figure 5B**). Under 2022-irrigated environment, two genotype groups were separated along the PC2 axis, explaining 30.9% of the variation. Most Group-A genotypes distributed in the positive direction of PC2, indicating low values of TRG (eigenvectors on PC1 and PC2: -0.60 and -0.52) and diosgenin content (eigenvectors on PC1 and PC2: 0.43 and -0.85) in these genotypes, while Group-B genotypes such as Çiftçi/TR, Sivas/TR, Konya/TR, Amasya/TR and Samsun/TR showed positive correlations with high TRG and diosgenin (**Figure 5C**). Furthermore, among these three studied bioactive compounds, diosgenin displayed a significant negative correlation with TRG and 4-HIL under 2021-irrigated (**Figure 6A**) and 2022-non-irrigated (**Figure 6B**), respectively while TRG was found to be positively correlated with 4-HIL under 2022-irrigated environment (**Figure 6C**). This indicated that the changing climate and irrigation pattern influenced these compounds differently. Thus, they are likely to be controlled by separate genetic mechanisms.

### 3.3 Stability of genotypes in bioactive compounds across environments

**Figure 7:**
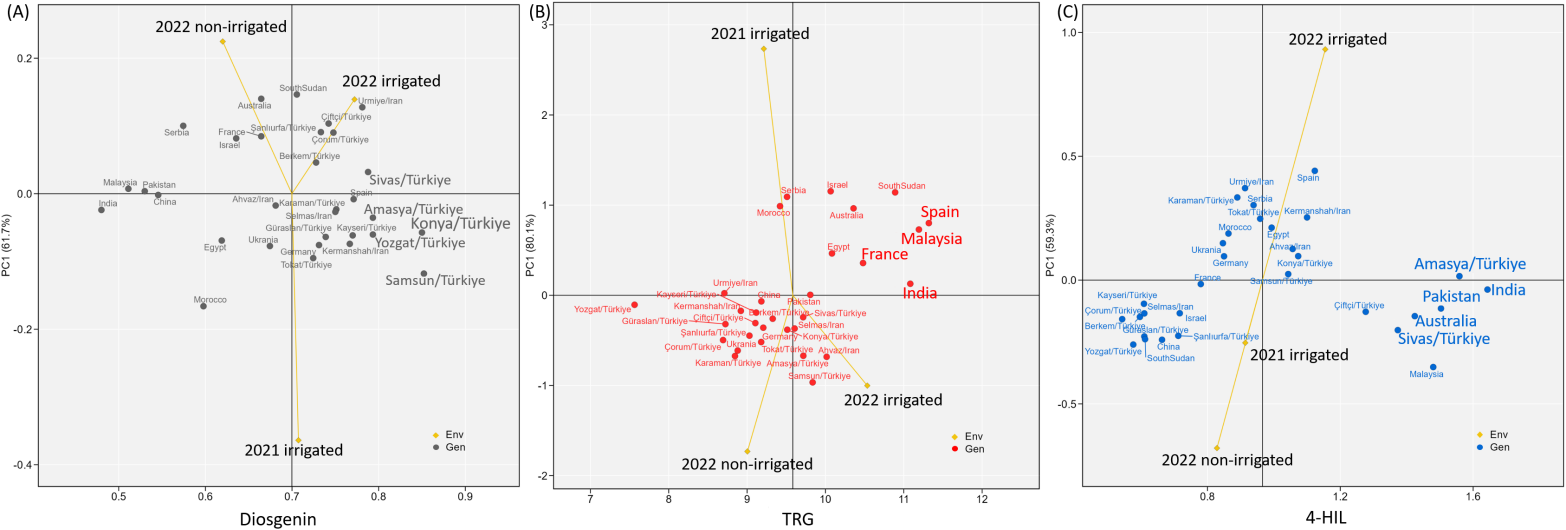
Additive main effects and multiplicative interaction (AMMI) biplots showing **(A)** diosgenin, **(B)** TRG and **(C)** 4-HIL versus the first principal component (PC1) score of 31 genotypes and three environments including 2021-irrigated, 2022-non-irrigated and 2022-irrigated. Genotypes located closer to the horizontal axis have higher stability across three environments while the vertical axis indicates the mean value of the 31 genotypes.

The additive main effects and multiplicative interaction (AMMI) results showed a contrasting adaptability pattern of the 31 genotypes in diosgenin, TRG and 4-HIL. The high and stable diosgenin contents were found in Group B genotypes i.e. Sivas/TR, Amasya/TR, Konya/TR, Yozgat/TR and Samsun/TR (**Figure 7A**), while all the high and stable TRG contents were identified in Group A genotypes i.e. Spain, Malaysia, France and India (**Figure 7B**). No clear regional pattern was found for genotypes with high and stable 4-HIL content (**Figure 7C**).

## 4 Discussion

The genetic structure of a plant is a fundamental factor in determining the synthesis and quantity of bioactive compounds. Different genotypes, owing to their unique metabolic pathways, can significantly influence the concentrations of these compounds, even within the same species. However, the regulation of bioactive compound synthesis is not solely dictated by genetics; environmental factors also play a crucial role. Temperature, rainfall, and light availability can substantially impact the levels of these compounds, either promoting or inhibiting their production (Khare et al., 2020; Mansinhos et al., 2024). While the genetic makeup establishes baseline production potential, environmental factors can modulate this potential, either enhancing or reducing the content of these compounds. Therefore, the interaction between genotype and environment is crucial, as it integrates these factors to shape the biosynthetic outcomes, determining which bioactive compounds are more prominently produced under a specific condition.

The impact of genotype-environment interactions becomes particularly evident when comparing fenugreek genotypes across different environmental conditions. The significant variations in compound concentrations observed among different fenugreek genotypes under varying environmental conditions can be attributed to genetic factors, environmental influences, and interactions. Genetic variability plays a critical role, with some genotypes exhibiting resilience to environmental stress, maintaining stable levels of bioactive compounds such as phenolics and antioxidants despite fluctuations in temperature, soil quality, and moisture (Singh et al., 2013). This genetic stability is often associated with specific alleles that enhance the synthesis of beneficial compounds under stress (Zandi et al., 2015). Conversely, other genotypes show significant variations in their chemical profiles due to their sensitivity to environmental changes, which may trigger or inhibit the biosynthetic pathways responsible for producing key phytochemicals (Güzel and Özyazıcı, 2021). For example, some genotypes increase polyphenol production under high temperatures or limited light, while others do not respond similarly. Additionally, genotype-environment interactions (G×E) significantly impact the phenotypic expression of compounds, as certain genotypes thrive in specific climatic and soil conditions while others perform poorly (Maurya et al., 2023; Camlica and Yaldiz, 2024; Coban et al., 2024). This interaction underscores the importance of selecting genotypes with favorable traits for specific growing conditions to optimize fenugreeks nutritional and medicinal potential. For instance, genotypes adapted to semiarid climates may yield higher quality and quantity of bioactive compounds compared to those grown in humid environments, where they may be more susceptible to stress and disease (Zandi et al., 2015).

### 4.1 Diosgenin shows significant variability due to both genetic and environmental differences

Diosgenin, a steroidal saponin, has been extensively studied for its pharmacological applications, especially due to its cholesterol-lowering and anti-inflammatory properties (Pari et al., 2012; Tak et al., 2024). However, research on how agronomic practices affect diosgenin content in fenugreek remains relatively scarce. Several studies have documented significant variability in diosgenin levels among different genotypes, underscoring the impact of genetic differences on its content.

Indeed, previous studies have reported a wide range of diosgenin concentrations across various fenugreek genotypes, further highlighting the genetic influence on its accumulation. Provorov et al. (1996) reported diosgenin concentrations across 31 different fenugreek genotypes, with the lowest level found in a Ukrainian genotype at 1.14% and the highest in a Polish genotype at 1.64%. In contrast, Król-Kogus et al. (2018) reported lower diosgenin levels of 0.18% in a Polish genotype and 0.13% in an Algerian genotype, values significantly below those observed in our study. Other research has also highlighted variability in diosgenin content: Taylor et al. (2000) found levels ranging from 0.38 to 0.79%, while Taylor et al. (2002) reported a range of 0.32 to 0.64%. Similarly, Lee (2009) documented diosgenin concentrations between 0.50 and 0.81%, Kaufmann et al. (2007) reported values from 0.097 to 0.159%, and Laila et al. (2014) observed levels ranging from 0.113 to 0.135%. Additional studies by Giridhar et al. (2016) and Saxena et al. (2017) reported diosgenin contents between 0.52 to 0.97% and 0.35 to 0.78%, respectively. On the other hand, Beyzi et al. (2021), in their study involving the Güraslan, which is also included in our research, reported that the diosgenin content ranged between 0.43 and 0.52%. These findings are consistent with the results of our study, suggesting that genetic differences among fenugreek genotypes play a crucial role in determining diosgenin content. This variability highlights the importance of selecting appropriate genotypes for optimizing diosgenin production, especially in the context of breeding and cultivation strategies aimed at enhancing the medicinal value of fenugreek.

Beyond genetic factors, environmental conditions also play a crucial role in diosgenin accumulation, particularly under stress conditions such as drought. In our study, a significant decrease in diosgenin levels was observed under drought stress (2022-irrigated: 0.77% *vs*. 2022-non-irrigated: 0.62%), attributed to the combined effects of multiple factors, including alterations in gene expression and enzyme activity, disruptions in cellular osmotic balance, energy metabolism changes, and oxidative stress (Javan et al., 2024). Under drought conditions, the expression levels of certain key genes involved in diosgenin biosynthesis may be reduced, negatively impacting the biosynthetic process. Key genes such as CYP90B, CYP94, and CYP72A, which are part of the cytochrome P450 enzyme family, play critical roles in diosgenin production. Additionally, genes like HMGCS (hydroxymethylglutaric acid CoA synthetase), MVK (mevalonate kinase), and squalene epoxidase (SE) are essential for converting precursors into diosgenin. The downregulation of these genes under drought stress may significantly hinder diosgenin accumulation (Sun et al., 2017; Li et al., 2022).

Furthermore, specific enzymes that facilitate diosgenin biosynthesis are also affected by drought stress, contributing to reduced accumulation. Cycloartenol synthase (CAS) and beta-glucosidase (BG) play essential roles in diosgenin biosynthesis. CAS catalyzes a critical step by converting squalene epoxide into cycloartenol, which is a precursor for steroidal saponins, including diosgenin. BG, on the other hand, is necessary for converting diosgenin from its glycosidic form into free diosgenin. Under drought stress, decreased expression levels of these enzymes can lead to a reduction in diosgenin production. Studies on fenugreek have indeed shown that drought stress lowers the expression of these genes involved in diosgenin biosynthesis (Javan et al., 2024; Maleki et al., 2024).

Conversely, under irrigated conditions, diosgenin production appears to be more favorable due to improved plant growth and metabolic efficiency. Irrigation conditions provide a more favorable environment for plant growth and development. Therefore, higher yield and improved yield components are expected outcomes (Ali et al., 2023; Guo et al., 2023). Lee (2009) reported that steroidal saponins in fenugreek are localized as furostanol glycosides within the cell walls of the embryo, and smaller seeds generally have lower diosgenin levels. In line with this, the higher diosgenin content observed in seeds grown under irrigated conditions in our study is related to these findings.

A similar pattern was observed for TRG biosynthesis, which also exhibited a decline under drought stress. TRG biosynthesis and accumulation also showed a decline under drought stress, reflecting the complex metabolic and physiological adaptations of plants. During drought stress, plants reorganize their metabolic priorities to survive, leading to reduced production of certain secondary metabolites (Farooq et al., 2009). Since the synthesis of secondary metabolites requires substantial energy, limited energy availability under drought conditions forces plants to deprioritize these energy-intensive processes, resulting in a decrease in some bioactive compounds (Chaves et al., 2003; Jaleel et al., 2009).

### 4.2 Trigonelline synthesis is restricted under drought, and it may be competing with diosgenin

TRG, a nitrogenous compound, plays a role in amino acid metabolism, but under drought stress, its synthesis may be restricted as plants channel energy and resources toward more critical processes, such as the production of compatible solutes (e.g., proline, glycinebetaine, and trigonelline), maintaining osmotic balance, and preventing water loss (Ashihara et al., 2015). Although the need to maintain osmotic balance promotes the synthesis of osmoregulators like proline and betaine, TRG production may be deprioritized. Furthermore, increased levels of abscisic acid (ABA) during drought stress cause stomata to close to reduce water loss, which can also inhibit the synthesis of secondary metabolites, thereby reducing TRG levels (Salvi et al., 2021).

Indeed, variations in TRG content depending on ecological conditions and crop years have been reported (Baghbani-Arani et al., 2017; Salehi et al., 2019). In our study, a wide variation in total TRG content (5.2–13.0 mg/g) was detected. These values are generally consistent with findings from previous research; however, significant differences were observed based on genotype, cultivation practices, and ecological factors. Such variability underscores the strong influence of environmental conditions on TRG accumulation, as reported in multiple studies. For example, Camlica and Yaldiz (2024) reported TRG content for fenugreek genotypes grown under two distinct environmental conditions in Northwestern Türkiye. Under irrigated conditions, TRG content ranged from 2.3 to 4.6 mg/g, while under dryland conditions, it ranged from 2.4 to 4.8 mg/g​. Beyzi et al. (2021) conducted their study in Central Anatolia, Türkiye, under irrigated conditions with varying phosphorus fertilizer levels, documenting TRG values between 7.4 and 9.7 mg/g​. Similarly, Güzel and Özyazıcı (2021) explored fenugreek grown in semiarid Southeastern Anatolia, Türkiye, noting TRG levels between 7.1 and 13.2 mg/g​. On the other hand, Bakhtiar et al. (2024) studied wild fenugreek species under uniform cultivation conditions at Iran, and reported TRG levels ranging from 4.26 to 6.78 mg/g​.

Beyond environmental influences, metabolic trade-offs may also explain fluctuations in TRG content, particularly in relation to diosgenin levels. The negative correlation observed between diosgenin and trigonelline in fenugreek likely arises from the interplay of competing biosynthetic pathways and adaptive responses to environmental stress. Diosgenin, a steroidal saponin synthesized via the mevalonate pathway, and trigonelline, an alkaloid derived from the shikimic acid pathway, may compete for shared substrates or enzymatic resources within the plant, leading to a trade-off in their synthesis (Naika et al., 2022). This trade-off is a common phenomenon in plants, where metabolic resources are allocated dynamically based on environmental and physiological needs. Additionally, this negative correlation may reflect an adaptive response to stress, as both compounds play crucial roles in plant defense mechanisms. Diosgenin has been linked to the regulation of oxidative stress through pathways such as Nrf2 and AMPK, while trigonelline has shown protective effects against oxidative damage and improved insulin sensitivity in diabetic models (Li et al., 2019; Tang et al., 2024). Under specific stress conditions, the plant may prioritize the synthesis of one compound over the other depending on the type of stress encountered, leading to variations in their concentrations (Mahmoudi et al., 2021).

Furthermore, genetic variability among fenugreek genotypes can significantly influence this relationship, as certain genotypes may exhibit regulatory mechanisms that favor the production of one compound over the other under environmental conditions (Naika et al., 2022). This highlights the importance of genotype selection in optimizing bioactive compound production, particularly in breeding programs aimed at enhancing fenugreek’s medicinal and nutritional potential. Understanding these interactions provides valuable insights into the metabolic regulation of fenugreek and offers opportunities to optimize its nutritional and medicinal potential.

### 4.3 Fenugreek produces higher 4-hydroxyisoleucine levels under irrigated conditions

The analysis also revealed that the amount of 4-HIL in fenugreek genotypes was higher under irrigated conditions compared to non-irrigated conditions. 4-HIL is synthesized through the modification of L-isoleucine, a process influenced by various enzymes whose activity can vary based on the plant’s physiological state and environmental factors (Yang et al., 2021). This compound plays a significant role in carbon and nitrogen metabolism and can influence energy production pathways and protein synthesis, thereby supporting overall plant growth and development (Lai et al., 2022).

However, drought stress significantly alters the metabolic priorities of plants, which in turn affects 4-HIL biosynthesis. During drought stress, as a survival and stress management strategy, plants redirect their energy and resources toward essential metabolic processes. This reallocation results in decreased synthesis of secondary metabolites, particularly nitrogenous compounds. The biosynthesis of 4-HIL is thought to be diminished as a result of this metabolic shift (Hura et al., 2022).

These variations in 4-HIL content across different environmental conditions have also been observed among various fenugreek genotypes. Fenugreek seeds contain varying levels of 4-HIL, an amino acid derivative known for its antidiabetic properties. In our study, the highest 4-HIL levels were observed in 2022 under both irrigated (1.90%) and rainfed (1.82%) conditions in the Indian genotype. Previous research highlights the significant influence of different genotypes and environmental conditions on the concentration of this compound. For instance, a wide range of 4-HIL levels has been reported across different studies, emphasizing the role of genetic and environmental factors. Haeri et al. (2011) reported 0.27% 4-HIL in Iranian genotypes, while Gopu et al. (2008) found concentrations ranging between 0.207% and 0.259% in Indian genotypes. Similarly, Singh et al. (2025) reported 0.55% in yellow-seeded fenugreek and 0.81% in green-seeded fenugreek in an Indian genotype, while Haxhiraj et al. (2024) indicated that the 4-HIL levels could vary between 1% and 2% in certain samples.

Beyond genetic and environmental influences, post-harvest factors also play a crucial role in determining the final 4-HIL content in fenugreek. The 4-HIL content in fenugreek seeds is influenced not only by genetic factors but also by environmental conditions such as the growth environment, soil composition, climate, and agricultural practices (Gopu et al., 2008; Haeri et al., 2011). Additionally, post-harvest drying and storage conditions play a crucial role in the stability and concentration of this compound (Al Mosawi, 2021). Notably, certain cultivars selected for medicinal purposes are known to have higher levels of 4-HIL (Gopu et al., 2008). Therefore, optimizing both cultivation and post-harvest strategies is essential to ensure consistent and high-quality 4-HIL production in fenugreek. Considering these factors, the selection of fenugreek varieties for medicinal or industrial purposes should carefully account for genetic makeup, cultivation conditions, and post-harvest processes to ensure the desired levels of bioactive compounds.

To further refine genotype selection, statistical tools such as PCA and cluster analysis have been widely employed to assess genetic diversity and adaptability. Many studies conducted under different environmental conditions have shown that PCA effectively reveals morphological differences among genotypes and guides the selection of superior genotypes. Complementing this, cluster analysis groups genotypes based on similar characteristics, facilitating the identification of genetic diversity and playing an integral role in understanding G×E interactions. The integration of PCA and cluster analysis has been widely employed to reveal morphological differences, genetic diversity, and adaptability among the fenugreek genotypes. Studies by Maleki et al. (2021), Hasaroeih et al. (2023), and Alqathama et al. (2024) highlighted the role of PCA and cluster analysis in identifying superior genotypes. This approach has been particularly useful in research, as demonstrated by Camlica and Yaldiz (2024), who found significant differences in morphology, yield, and bioactive properties among genotypes, emphasizing their adaptability to diverse environmental conditions.

## 5 Conclusion

The study has revealed significant findings on diosgenin, TRG, and 4-HIL; these compounds exhibited notable variations across genotypes under both irrigated and non-irrigated conditions. The diosgenin content in Group B genotypes was generally higher than in other genotypes, highlighting its potential in the development of pharmaceutical products due to its cholesterol-lowering and anti-inflammatory properties. The results demonstrated that variations in bioactive compound levels were more pronounced under different environmental conditions, and higher yields were typically observed under irrigated conditions. Due to its suitability for mechanization, short growing season, and lower costs, fenugreek cultivation is regarded as a more suitable alternative for diosgenin production compared to traditional methods involving plants from the Dioscorea family. These advantages present the potential to further increase the agricultural and pharmacological value of fenugreek.

In terms of breeding, selecting bioactive compound-stable genotypes is of great interest, especially in the context of climate change. The broad genetic background of genotypes studied here allowed the search for superior genetic resources in these bioactive compounds. Interestingly, a clear genotype group pattern was identified for high and stable bioactive compounds. Genotypes with high and stable diosgenin were all group-B genotypes. The high and stable TRG was only found in group-A genotypes while the high and stable 4-HIL was found in genotypes from both groups. This trend further confirmed the different genetic mechanisms underlying these three bioactive compounds. The Turkish genotypes such as Sivas, Amasya, Konya, Yozgat and Samsun can be used as high-diosgenin germplasms in future breeding, while the genotypes Spain, Malaysia, France and India were screened out with promising potential for high TRG content. More studies are needed to dissect the genetic background of high 4-HIL content.

Future studies should also incorporate transcriptomic and metabolomic approaches to validate the observed trends in bioactive compound synthesis and identify the genetic and molecular pathways regulating these compounds. Such integrative analyses will help refine breeding strategies for fenugreek and enhance its value as a pharmacological and agricultural crop.

## Data Availability

The raw data supporting the conclusions of this article will be made available by the authors, without undue reservation.
