# Supplementary material for: Characterization of bioactive compounds in fenugreek genotypes in varying environments: diosgenin, trigonelline, and 4-hydroxyisoleucine
Source: Front Plant Sci. 2025 Mar 24;16:1562931. doi: 10.3389/fpls.2025.1562931 (PMC11973278; doi:10.3389/fpls.2025.1562931)
Supplement: Supplementary file 1 [file Table1.docx]

**SUPPLEMENTARY TABLE**

**Supplementary Table 1** The average values of diosgenin content (%), trigonelline content (mg g^-1^), and 4-hydroxyisoleucine content (%) for different fenugreek genotypes under 2021 irrigated, 2022 non-irrigated, and 2022 irrigated conditions.

| **Genotype** | **Diosgenin Content (%)** | | | **Trigonelline Content (mg g-1)** | | | **4-hydroxyisoleucine Content (%)** | | |
| --- | --- | --- | --- | --- | --- | --- | --- | --- | --- |
|  | **2021** | **2022** | | **2021** | **2022** | | **2021** | **2022** | |
|  | **Irrigated** | **Non-irrigated** | **Irrigated** | **Irrigated** | **Non-irrigated** | **Irrigated** | **Irrigated** | **Non-irrigated** | **Irrigated** |
| Indıa | 0,50±0,01 m | 0,42±0,03 l | 0,52±0,01 k | 10,88±0,20 e | 9,35±0,11 a-f | 13,02±0,02 a | 1,21±0,02 d | 1,82±0,04 a | 1,90±0,11 a |
| Pakistan | 0,54±0,01 kl | 0,47±0,04 ı-l | 0,58±0,01 jk | 9,57±0,21 f | 9,83±0,17 a-e | 10,02±0,02 ıjk | 1,35±0,03 c | 1,54±0,03 abc | 1,62±0,08 bcd |
| Serbia | 0,55±0,02 k | 0,54±0,02 g-j | 0,63±0,01 ıjk | 11,77±0,44 d | 5,22±0,04 h | 11,54±0,14 cd | 0,89±0,03 gh | 0,54±0,03 ghı | 1,39±0,08 efg |
| Israel | 0,62±0,03 j | 0,59±0,02 d-h | 0,70±0,07 e-j | 12,90±0,53 bc | 7,74±0,11 fg | 9,56±0,06 mn | 0,74±0,03 ı | 0,64±0,04 e-ı | 0,77±0,05 klm |
| Egypt | 0,66±0,01 ı | 0,55±0,01 f-ı | 0,65±0,01 h-k | 11,14±0,17 e | 9,51±0,06 a-e | 9,60±0,14 lmn | 0,47±0,01 o | 1,02±0,08 d | 1,49±0,11 c-f |
| Malaysia | 0,52±0,02 lm | 0,44±0,01 kl | 0,58±0,03 jk | 12,76±0,58 bc | 9,06±0,12 a-f | 11,76±0,08 cd | 1,41±0,06 b | 1,66±0,09 ab | 1,37±0,14 efg |
| Ukrania | 0,71±0,03 gh | 0,58±0,03 e-h | 0,74±0,02 c-ı | 6,87±0,26 no | 9,55±0,18 a-e | 10,22±0,04 hı | 0,85±0,03 h | 0,54±0,01 ghı | 1,15±0,08 hıj |
| Germany | 0,77±0,02 ef | 0,64±0,02 b-g | 0,79±0,02 a-h | 7,93±0,17 h-k | 9,64±0,06 a-e | 10,05±0,08 ıj | 0,92±0,02 g | 0,54±0,03 ghı | 1,09±0,06 ıj |
| Morocco | 0,66±0,01 ı | 0,45±0,02 jkl | 0,69±0,05 f-j | 12,03±0,26 d | 8,55±0,16 c-g | 7,68±0,03 p | 1,02±0,02 f | 0,41±0,01 ı | 1,16±0,05 hı |
| China | 0,55±0,01 kl | 0,42±0,03 kl | 0,67±0,06 g-k | 8,63±0,14 g | 8,72±0,11 a-g | 10,20±0,09 hı | 0,74±0,01 ı | 0,64±0,03 e-ı | 0,61±0,04 mno |
| Australia | 0,62±0,01 j | 0,61±0,02 d-h | 0,76±0,06 b-ı | 12,55±0,24 c | 7,71±0,07 fg | 10,82±0,06 fg | 1,36±0,03 bc | 1,42±0,03 bc | 1,49±0,10 c-f |
| Spain | 0,78±0,01 cde | 0,68±0,01 a-d | 0,85±0,06 a-d | 13,15±0,25 ab | 9,41±0,20 a-f | 11,40±0,14 de | 1,20±0,02 d | 0,51±0,04 hı | 1,66±0,09 bc |
| South Sudan | 0,66±0,02 ı | 0,64±0,01 b-g | 0,82±0,03 a-f | 13,65±0,36 a | 8,38±0,06 d-g | 10,64±0,01 g | 0,62±0,02 lm | 0,64±0,04 e-ı | 0,58±0,02 mno |
| France | 0,64±0,01 ıj | 0,60±0,03 d-h | 0,76±0,07 b-ı | 11,04±0,19 e | 9,04±0,11 a-f | 11,36±0,04 de | 0,71±0,01 ıj | 0,67±0,06 e-ı | 0,96±0,05 jk |
| Selmas/Iran | 0,77±0,01 der | 0,68±0,03 a-d | 0,80±0,03 a-g | 8,38±0,09 gh | 10,44±0,17 a | 10,00±0,17 ı-l | 0,72±0,01 ıj | 0,47±0,05 ı | 0,64±0,03 mno |
| Urmiye/Iran | 0,74±0,01 fg | 0,72±0,02 abc | 0,89±0,05 ab | 8,56±0,16 g | 8,90±0,11 a-f | 8,67±0,04 o | 0,61±0,01 lm | 0,64±0,05 e-ı | 1,49±0,02 c-f |
| Ahvaz/Iran | 0,70±0,02 h | 0,63±0,02 c-g | 0,72±0,02 d-j | 7,66±0,21 jkl | 9,99±0,18 a-d | 12,40±0,29 b | 0,87±0,02 gh | 0,91±0,02 de | 1,39±0,07 efg |
| Kermanshah/Iran | 0,80±0,02 bcd | 0,66±0,03 b-e | 0,84±0,03 a-e | 8,20±0,21 ghı | 9,27±0,13 a-f | 9,29±0,34 n | 1,23±0,03 d | 0,61±0,03 f-ı | 1,46±0,04 def |
| Konya/Türkiye | 0,88±0,03 a | 0,77±0,04 a | 0,90±0,02 ab | 8,00±0,27 hıj | 9,09±0,17 a-f | 11,45±0,14 de | 1,12±0,04 e | 0,78±0,03 d-h | 1,32±0,03 fgh |
| Samsun/Türkiye | 0,90±0,04 a | 0,73±0,01 ab | 0,93±0,02 a | 6,70±0,29 o | 10,29±0,10 abc | 12,52±0,07 b | 1,08±0,05 e | 0,82±0,05 d-g | 1,23±0,07 ghı |
| Yozgat/Türkiye | 0,82±0,01 b | 0,68±0,01 a-d | 0,88±0,02 abc | 6,89±0,06 no | 7,09±0,10 g | 8,71±0,08 o | 0,64±0,01 kl | 0,58±0,04 ghı | 0,51±0,06 o |
| Amasya/Türkiye | 0,81±0,01 bc | 0,68±0,04 a-d | 0,89±0,02 ab | 7,42±0,13 lm | 9,84±0,12 a-e | 11,89±0,07 c | 1,63±0,03 a | 1,32±0,05 c | 1,73±0,04 ab |
| Karaman/Türkiye | 0,77±0,01 def | 0,68±0,06 a-d | 0,80±0,04 a-g | 6,77±0,08 o | 10,11±0,11 a-d | 9,66±0,13 j-n | 0,64±0,01 kl | 0,61±0,03 f-ı | 1,42±0,03 efg |
| Sivas/Türkiye | 0,78±0,01 cde | 0,69±0,03 a-d | 0,89±0,05 ab | 8,57±0,14 g | 8,98±0,06 a-f | 11,60±0,07 cd | 1,35±0,02 c | 1,39±0,05 bc | 1,38±0,13 efg |
| Şanlıurfa/Türkiye | 0,71±0,03 gh | 0,69±0,01 a-d | 0,80±0,04 a-g | 7,32±0,29 lmn | 8,64±0,09 b-g | 11,13±0,01 ef | 0,84±0,03 h | 0,64±0,06 e-ı | 0,66±0,09 l-o |
| Çorum/Türkiye | 0,72±0,01 gh | 0,67±0,02 a-e | 0,85±0,02 a-d | 7,01±0,13 mno | 9,21±0,10 a-f | 9,86±0,08 ı-m | 0,59±0,01 lm | 0,47±0,04 ı | 0,57±0,02 no |
| Tokat/Türkiye | 0,77±0,01 def | 0,64±0,02 b-g | 0,76±0,02 b-ı | 7,49±0,14 klm | 10,00±0,07 a-d | 10,05±0,22 ıj | 0,54±0,01 n | 0,88±0,08 def | 1,46±0,03 def |
| Kayseri/Türkiye | 0,80±0,02 bcd | 0,68±0,04 a-e | 0,83±0,03 a-f | 8,29±0,22 gh | 9,22±0,09 a-f | 9,84±0,13 ı-m | 0,58±0,02 mn | 0,54±0,04 ghı | 0,71±0,0 lmn |
| Berkem/Türkiye | 0,72±0,01 gh | 0,67±0,02 a-e | 0,80±0,01 a-g | 8,25±0,07 gh | 9,22±0,10 a-f | 10,51±0,12 gh | 0,61±0,01 lm | 0,54±0,07 ghı | 0,64±0,09 mno |
| Çiftçi/Türkiye | 0,71±0,01 gh | 0,67±0,02 a-e | 0,84±0,04 a-e | 7,71±0,13 ı-l | 8,11±0,09 efg | 11,50±0,36 cde | 1,13±0,02 e | 1,32±0,09 c | 1,38±0,06 efg |
| Güraslan/Türkiye | 0,77±0,01 def | 0,65±0,01 b-f | 0,80±0,02 a-g | 7,54±0,13 jkl | 9,01±0,07 a-f | 9,62±0,08 k-n | 0,68±0,01 jk | 0,58±0,01 ghı | 0,57±0,07 no |
| **Mean** | **0,71±0,02** | **0,62±0,02** | **0,77±0,03** | **9,21±0,22** | **9,06±0,11** | **10,55±0,17** | **0,91±0,02** | **0,83±0,04** | **1,16±0,07** |

Means within the same column followed by different letters indicate statistically significant differences (p < 0.05).
